# How does curcumin work with poor bioavailability? Clues from experimental and theoretical studies

**DOI:** 10.1038/srep20872

**Published:** 2016-02-18

**Authors:** Liang Shen, Cui-Cui Liu, Chun-Yan An, Hong-Fang Ji

**Affiliations:** 1 Shandong Provincial Research Center for Bioinformatic Engineering and Technique, School of Life Sciences, Shandong University of Technology, Zibo 255049, P. R. China

## Abstract

Curcumin is a natural product with multiple biological activities and numerous potential therapeutic applications. However, its poor systemic bioavailability fails to explain the potent pharmacological effects and hinders its clinical application. Using experimental and theoretical approaches, we compared curcumin and its degradation products for its biological activities against Alzheimer’s disease (AD), including the superoxide anion radical (O_2_^.–^)-scavenging activity, Aβ fibrils (fAβ) formation-inhibiting activity, and enzymatic inhibition activity. We showed that compared to the parent compound curcumin, the degradation products mixture possessed higher O_2_^.–^-scavenging activity and stronger inhibition against fAβ formation. The docking simulations revealed that the bioactive degradation products should make important contribution to the experimentally observed enzymatic inhibition activities of curcumin. Given that curcumin is readily degraded under physiological condition, our findings strongly suggested that the degradation products should make important contribution to the diverse biological activities of curcumin. Our novel findings not only provide novel insights into the complex pharmacology of curcumin due to its poor bioavailability, but also open new avenues for developing therapeutic applications of this natural product.

Curcumin (1,7-bis(4-hydroxy-3-methoxyphenyl)-1,6-heptadiene-3,5-dione, Fig. 1), is a polyphenol isolated from the Indian spice turmeric and has been used as a traditional medicinal agent in Ayurvedic medicine for thousands of years. Curcumin has received considerable attention over the past decades, which is mainly due to its diverse biological activities, including antioxidant, anti-inflammatory, antiarthritic, and antibacterial activities, and its potential therapeutic applications in a large number of diseases such as cancer and neurodegenerative diseases^1^,^2^,^3^,^4^,^5^,^6^. Numerous preclinical and clinical studies indicated the great potential of curcumin in treating these diseases, but the application of curcumin in the therapeutic treatment was hindered by its poor systemic bioavailability^7^,^8^,^9^. Multiple studies has shown that, even with high doses of curcumin, the levels of curcumin as well as its *in vivo* metabolites are extremely low in serum and tissues after a short period of time^7^,^10^,^11^. For instance, it has been reported that no curcumin was detected in serum 1, 2, and 4 hours after administration of a single oral dose of 500 to 8000 mg in human^11^. Similarly, after administration of 440–2200 mg/day of oral curcuma extract for up to 29 days to patients with advanced colorectal cancer, neither curcumin nor its *in vivo* metabolites were found in the plasma or urine of the subjects^10^. In addition, curcumin possesses ideal structure features as enzyme inhibitors, including a flexible backbone, hydrophobic nature, and several available hydrogen bond (H-bond) donors and acceptors, yet, as reviewed by Heger *et al.,* the experimental observed inhibitory activities of curcumin are much lower than those expected from its chemical structure^2^. These observations raise an intriguing question, that is, how curcumin is able to manifest remarkable biological effects under the condition of poor systemic bioavailability.

Curcumin has been proven to possess low stability in aqueous solution at physiological pH and degrades readily^12^,^13^,^14^,^15^. It was demonstrated that in phosphate buffer at pH 7.4, about 90% of curcumin degraded within 30 min^14^ and the degradation products have been identified as trans-6-(4′-hydroxy-3′-methoxyphenyl)-2,4-dioxo-5-hexenal, ferulic aldehyde, ferulic acid, feruloyl methane, vanillin, vanillic acid, and other dimerization end-products (Fig. 1)^2^,^14^,^15^. A recent *in vivo* study proved that selected degradation products mentioned above were the major human metabolites after curcumin consumption, and their levels were much higher than those of its metabolic compounds^16^. However, in terms of understanding the pharmacology of curcumin, the potential contribution of these degradation products has not gained enough attentions.

In this study, we have taken Alzheimer’s disease (AD)-associated therapeutic targets as examples and revealed an important contribution of curcumin degradation products to its biological activities by both experimental and theoretical approaches. The benefit effects of curcumin against AD are well supported by experimental, clinical and epidemiologic studies^17^,^18^,^19^,^20^,^21^,^22^,^23^. It is well known that the pathogenesis of AD involves multiple changes in the central nervous system, including increased oxidative stress, cholinergic deficit, increased amyloid-β peptide, and amyloid-β peptide fibrils (fAβ) formation. Using both experimental and theoretical approaches, we compared the superoxide anion radical (O_2_^.–^)-scavenging activities and fAβ formation-inhibiting activities of parent curcumin and its degradation products, and performed molecular docking calculations of parent curcumin, its *in vivo* metabolites and degradation products with AChE (acetylcholinesterase, an important target for AD therapeutic intervention to overcome the cholinergic deficit in AD), β-amyloid precursor cleavage enzyme (BACE-1, an enzyme important in producing amyloid-β peptide), and other model enzymes. The experimental results indicated that the degradation products mixture of curcumin possesses higher O_2_^.–^-scavenging and anti-fAβ formation activities than parent curcumin. The docking simulation results support that the bioactive degradation products should make important contribution to the experimentally observed inhibition of curcumin against these enzymes. All these findings pointed to the important contributions of degradation products to the diverse biological effects of curcumin.

## Results

### Degradation of curcumin

Our preliminary experiments and previous studies^14^,^15^ showed that curcumin degraded readily after incubated in phosphate buffered solutions (PBS) with high pH or temperature (data not shown). In order to compare the activities of parent curcumin and its degradation products mixture, a degradation condition (PBS, pH = 9.0, heated to 80 °C for 20 minutes) was employed to ensure the complete degradation of curcumin. Under this condition, curcumin degraded almost completely after 20 minutes and HPLC analysis showed that the degraded rate was 99.74% (Fig. 2).

### O_2_^.–^-scavenging activities of curcumin and its degradation products

To investigate the potential contribution of the degradation products to the biological effects of curcumin, we compared the O_2_^.–^-scavenging activity of curcumin and its degradation products mixture with the pyrogallol autoxidation method^24^. The IC_50_ value of the degradation products mixture was lower than that of curcumin as shown in Fig. 3. Thus, the degradation products mixture, as well as the single degradation compound (ferulic acid or vanillin) exhibited higher O_2_^**.–**^-scavenging ability than parent curcumin, despite relatively lower than L-ascorbic acid.

### Aβ fibrils formation-inhibiting activities of curcumin and its degradation products

Formation of fAβ is considered to be the central pathogenic event of AD. Many studies have reported that curcumin could inhibit the formation of fAβ^25^,^26^,^27^,^28^. Here, employing fluorescence spectroscopic analysis with thioflavin T (ThT) and electron microscopic, we examined whether the degradation products of curcumin also possess effect to inhibit the formation of fAβ(1–42). As shown in Fig. 4, ThT assays monitoring the formation of fAβ(1–42) indicated the inhibitory effect of curcumin and its degradation products mixture. Interestingly, the degradation products mixture also possessed obvious inhibitory effect of formation of fAβ(1–42). The decrease in ThT fluorescence with addition of the degradation products mixture was much more significant than that with parent curcumin, suggesting that the inhibitory effect of the degradation products mixture was greater than parent curcumin. Among the four groups, ferulic acid exhibited the highest inhibitory activity (Fig. 4).

After incubation with 20 μM fresh Aβ(1–42) at 37 °C for 6 hours, clear fibril growth was observed by electron microscopy (EM) as shown in Fig. 5A. However, the addition of curcumin inhibited the extension of fAβ(1–42), and few fibrils were observed occasionally (Fig. 5B). Interestingly, the degradation products mixture of curcumin also inhibited formation of fAβ(1–42) (Fig. 5C). Ferulic acid by itself significantly inhibited the extension of fAβ(1–42) (Fig. 5D). Together with a previous report showing that ferulic acid is able to destabilize preformed fAβ(1–42)^29^, our finding supports a strong inhibitory effect of ferulic acid, a degradation product, on fAβ(1–42) formation.

### Comparison of enzymatic inhibition by curcumin and its degradation products

In order to compare the inhibitory effects of curcumin and its degradation products on AD-realted AChE and BACE-1, we performed molecular docking study. First, we reviewed literature and found 8 enzymes that were experimentally inhibited by curcumin, which are AChE, BACE-1, HiV-1 protease (HiV-1 PR), HiV-2 protease (HiV-2 PR), Aldose reductase 1 (ALR1), Aldose reductase 2 (ALR2), cyclooxygenase-2 (COX-2) and sarco (endo) plasmic reticulum Ca^2+^-ATPase (SERCA). To ensure the accuracy of docking studies, all released crystal structures were employed as model systems.

**Figure 1:**
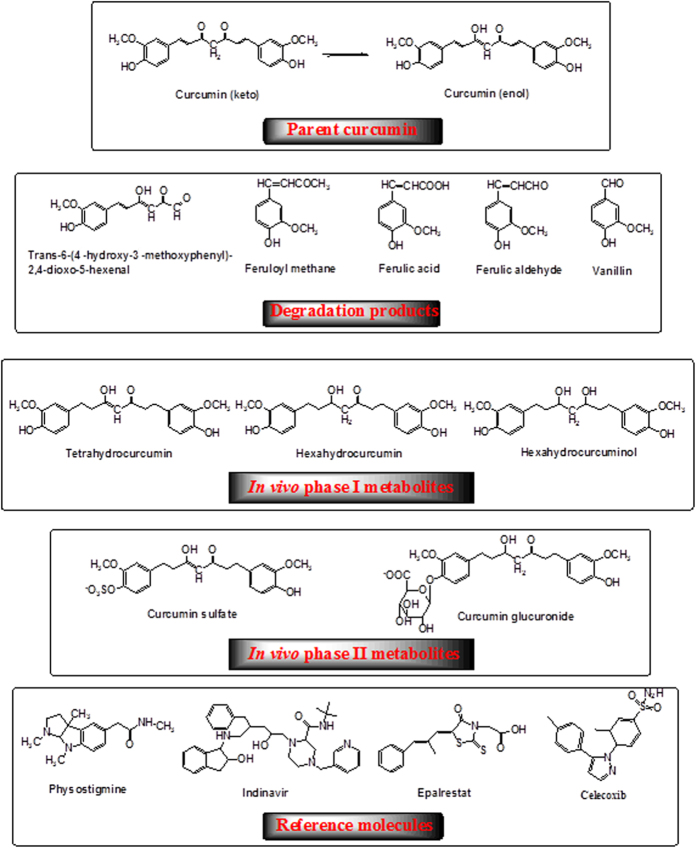
Chemical structures of curcumin, its degradation products, *in vivo* metabolites, and reference molecules.

**Figure 2:**
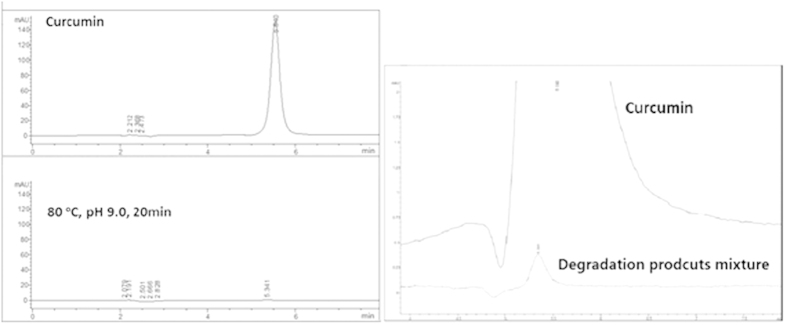
The HPLC analysis of curcumin and its degradation products mixture and the amplified figure. The samples were subjected to Agilent Extend-C18 column, with an eluting solution (MeOH: H_2_O: HAc = 75: 25: 0.5, 1.0 mL/min) and the detection wavelength of 430 nm.

**Figure 3:**
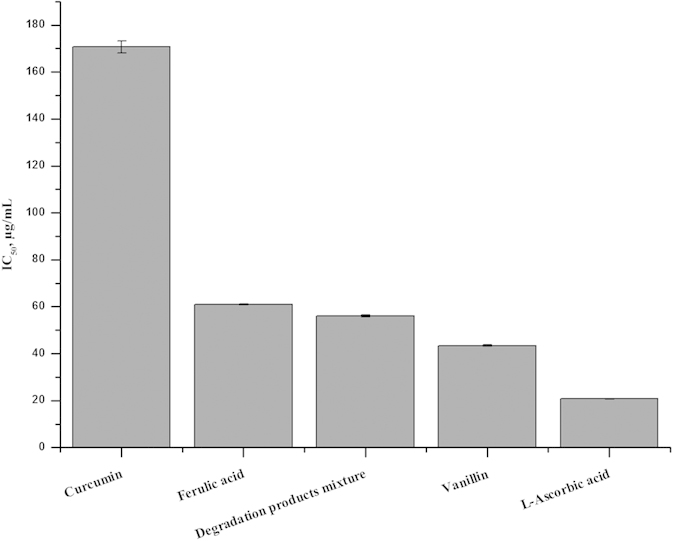
Superoxide-scavenging activity of curcumin, its degradation products mixture, ferulic acid, vanillin and L-ascorbic acid.

**Figure 4:**
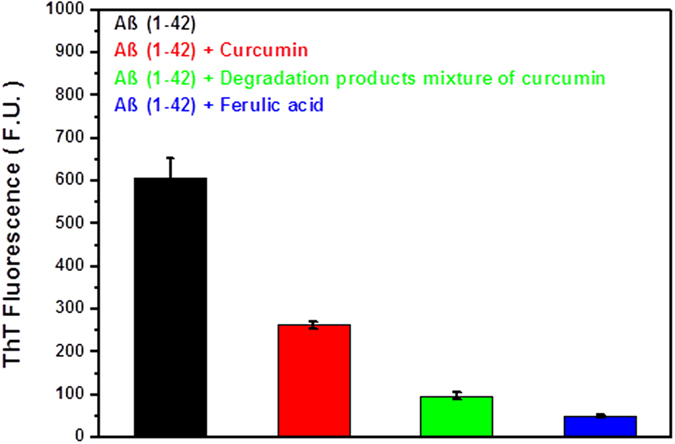
Inhibitive effects of human fAβ(1–42) formation by curcumin, its degradation products mixture and ferulic acid, evaluated by ThT fluorescence assay. Each volumes of ThT solution and Aβ(1–42) with PBS buffer, and curcumin or degradation products mixture of curcumin or ferulic acid were mixed in a 1:1:1 volume ratio, and then incubated at 37 °C for 6 h. The final concentrations of Aβ(1–42), ThT, curcumin, original concentration of curcumin to degrade, and ferulic acid were 20 μM.

**Figure 5:**
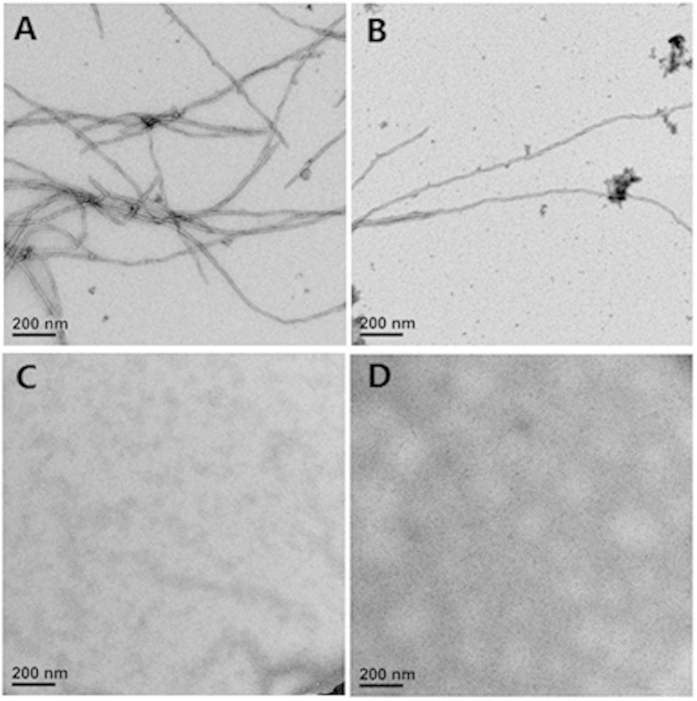
Electron microscopy imaging of human Aβ(1–42) fibrils when incubated alone (**A**), with curcumin (**B**), degradation products mixture of curcumin (**C**), or ferulic acid (**D**). Samples were dried and transferred into a Tecnai G2 Spirit BIOTWIN Electron microscope (FEI) operated at 120 kV. Images were acquired with a Gatan 895 4k x 4k CCD camera at a nominal magnification of 30,000 ×. Scale bars = 200 nm.

**Figure 6:**
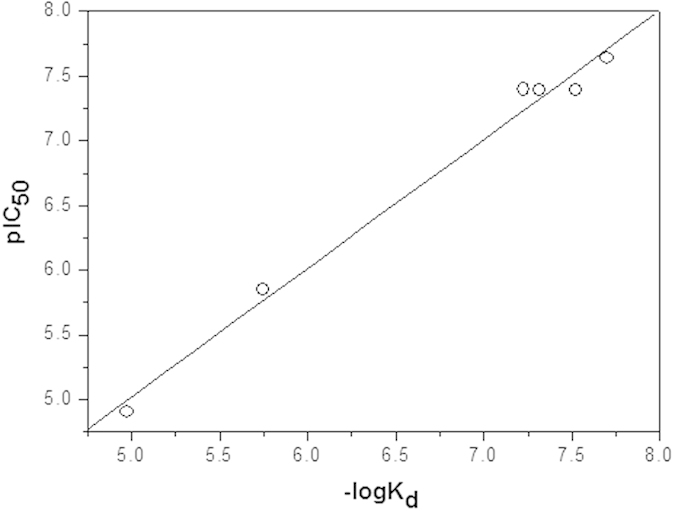
Correlation between pIC_50_ and −log_10_K_d_ of four reference molecules. Linear equation: pIC_50_ = 0.04 + 0.996 * (−log_10_K_d_) (*r* = 0.994, P < 0.0001).

To verify the accuracy of the employed docking methods, we first selected four known inhibitors for these enzymes (physostigmine, indinavir, epalrestat and celecoxib, Fig. 1) as reference molecules. We performed docking calculations and the results showed that binding modes of these four inhibitors to their respective host enzymes, including the interacting residues and forces contributing to binding, were consistent with previous docking studies^30^,^31^,^32^. Through Surflex-Dock calculations, the estimated –log_10_K_d_ of each reference molecules was calculated and listed in Table 1. There existed a good linear correlation between experimental pIC_50_^33^,^34^,^35^,^36^ and the calculated –log_10_K_d_ (equation 1, Fig. 6, details in the Methods), indicating that –log_10_K_d_ obtained from Surflex-Dock calculation was an appropriate theoretical parameter to characterize the enzyme-inhibiting activity in theoretical treatment of these systems.


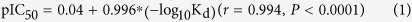


**Figure 7:**
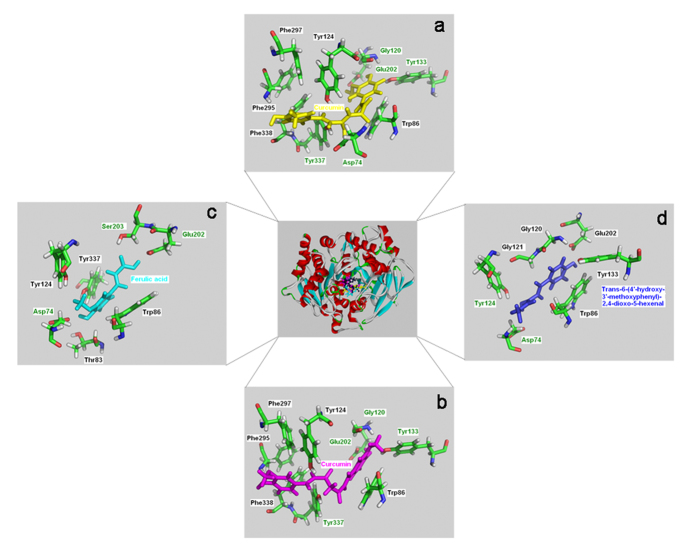
Close-up views of binding modes of human AChE with four curcuminoids. a-curcumin (enol form); b-curcumin (keto form); c-ferulic acid; d-trans-6-(4′-hydroxy-3′-methoxyphenyl)-2,4-dioxo-5-hexenal. The hydrogen bond interacting residues are labelled in green.

The same methods were employed to estimate the inhibitory activities of parent curcumin, its metabolites, and degradation products, for AChE, BACE-1, and other six enzymes. Curcumin exists in enol and keto forms in solution and both isomers of curcumin were docked into the eight enzymes^37^. According to the binding modes, curcumin could efficiently fit within the respective binding pockets of the eight enzymes. Figure 7 showed the binding modes of curcumin with human AChE and it can be seen that the interactions, including hydrogen bonds and hydrophobic interactions, were involved in the binding of curcumin with human AChE. Based on the theoretical –log_10_K_d_, the predicted IC_50_ of curcumin in inhibiting theses eight enzymes were calculated according to equation 1. However, the predicted IC_50_ for both the keto and enol forms of curcumin were hundreds of times lower than the reported experimental values for AChE, HiV-1 PR, HiV-2 PR, ALR1, ALR2, and SERCA, and tens of times lower than that for COX-2 (Table 2).

It has been shown that curcumin administered intraperitoneally or orally can undergo reduction to give birth to tetrahydrocurcumin, hexahydrocurcumin, octahydrocurcumin, curcumin glucuronide and curcumin sulfate (Fig. 1)^38^. Despite these metabolites also possess low serum and tissue bioavailability^7^,^10^, we performed parallel docking calculations on the metabolites to explore their possible contribution in enzymes inhibition. According to our docking calculations, the metabolites of tetrahydrocurcumin, hexahydrocurcumin and octahydrocurcumin also had significantly lower IC_50_ in comparison with the experimental values shown in Table 2. Curcumin glucuronide and curcumin sulfate showed no obvious inhibitory effects against these enzymes because of the steric effects.

As discussed above, curcumin possesses low stability and is degrade readily in aqueous solution at physiological pH^12^,^13^,^14^,^15^. The degradation products have been identified as trans-6-(4′-hydroxy-3′-methoxyphenyl)-2,4-dioxo-5-hexenal, ferulic aldehyde, ferulic acid, feruloyl methane, vanillin, vanillic acid, and other dimerization end-products (Fig. 1)^2^,^14^,^15^. Taking the poor bioavailability and low stability of curcumin into account, we asked the question whether the degradation products contribute to the observed inhibition for these enzymes.

According to parallel docking calculations of the degradation products with these enzymes, the general locations of the binding sites of the degradation products in each enzyme were similar to those of curcumin. For instance, as shown in Fig. 7, parent curcumin and the degradation products shared the same binding pockets in human AChE. In addition, the number of the interacting residues of the degradation products with enzymes decreased in comparison with that of parent curcumin (Fig. 7). The theoretical IC_50_ for these degradation products against the eight enzymes were shown in Table 2. Notably, the estimated IC_50_ for ferulic acid against AChE and ALR2 were close to the values determined in the experiments using ferulic acid as inhibitor^39^,^40^. Moreover, the IC_50_ for the degradation products were significantly lower to those of curcumin and its metabolites, and close to the experimental values for curcumin overall (Table 2). As curcumin degraded easily under these experimental conditions and different degradation products may have synergistic inhibitory effects, our results strongly suggested that the experimentally observed enzymatic inhibition by curcumin is mostly due to the activities of its bioactive degradation products.

## Discussion

Despite a wide range of pharmacological activities of curcumin reported in the past decades, a paradox remains regarding the pharmacology of curcumin owing to its physicochemical properties leading to the poor systemic bioavailability. Moreover, although nature endows curcumin ideal molecular functionalities as enzymes inhibitors, which include two hydrophobic phenyl domains connected by a flexible α, β-unsaturated β-diketo linker, and the phenolic and carbonyl functional groups located on the ends and at the center of the molecule to potentially participate in hydrogen bonding with target biomolecules (Fig. 1), numerous *in vitro* experiments indicated the low potency in enzyme inhibition^2^. The experimentally reported inhibitory activities of curcumin are much lower than those predicted based on its chemical structure^41^,^42^.

Low stability has been considered to be a hurdle for the clinical application of curcumin. Based on our experimental comparison of the O_2_^**.–**^-scavenging activities and fAβ(1–42) formation inhibiting activities of curcumin and its degradation products mixture and theoretical docking studies of the molecular mechanisms of enzyme inhibition of curcumin, we proposed that the degradation products curcumin are actually the main bioactive molecules in executing the biological activities of curcumin.

Our conclusion is consistent with previous observations. First, curcumin and its metabolites always have poor bioavailability *in vivo*, even with high doses^6^, however, its pharmacological activities have been widely recognized. Our finding provides a plausible explanation to the apparently contradictory observations. Second, it has been found that curcumin and its degradation products also possess similar pharmacological profiles in anti-cancer, anti-inflammation and antimicrobial activities, which is consistent with our conclusion that the bioactive degradation products of curcumin are important contributors to its pharmacological activities^2^,^43^,^44^. Third, a recent *in vivo* study showed that the degradation products aforementioned are the major human metabolites after curcumin consumption and their levels are much higher than those of curcuminoids^16^. Forth, when curcumin was added to inhibit lipoxygenase, the binding of selected degradation products rather than parent curcumin was proven by X-ray diffraction and mass spectrometry^45^, providing direct evidence supporting our theory.

## Conclusion

In summary, our novel experimental and theoretical findings suggested that the degradation products should play important roles in executing the biological and pharmacological activities of curcumin. Our finding not only provides a plausible explanation for the seemingly contradictory observations regarding biological activities of curcumin, it is also highly significant for the therapeutic application of this natural product against various human diseases.

## Methods

### Experimental methods

#### Materials

Curcumin (98%), ferulic acid (99%), vanillin (99%), L-ascorbic acid (98%), pyrogallol, human Aβ(1–42), ThT, Nutrient Mixture F-12 Ham, were obtained from the Sigma-Aldrich Shanghai Trading Co. (Shanghai, China); Tris(Hyroxymethyl)aminomethane (Tris) was obtained from Amresco Inc. (Solon, OH, USA). All other reagents were of analytical grade.

#### HPLC analysis

HPLC analysis was performed on an Agilent 1100 (USA) HPLC System equipped with an Agilent G1311A quaternary pump, an Agilent UV-DAD G1315B detector. Aliquots of 80 μL of curcumin stock solution (1.0 mg/mL, dissolved in methanol) were added to 1920 μL of 0.05 M PBS, pH = 9.0, and the sample was heated to 80 °C for 20 minutes. After incubation, the sample was acidified to pH = 7.0 with 6 N HCl. The incubated sample was filtrated through a 0.45 μm PVDF membrane filters. Then, 10.0 μL of the filtrated sample was injected and eluted with a mobile phase (MeOH: H_2_O: HAc = 25: 75: 0.5) and a flow rate of 1.0 mL/min at the detecting wavelength of 430 nm. The standard HPLC curve of curcumin was made with a curcumin solution prepared just before use to avoid degradation.

#### Superoxide-scavenging assay

The superoxide-scavenging activity of curcumin, its degradation products mixture, and the standard compounds was evaluated with an improved pyrogallol method, which improved the accuracy of the estimated activity as detailed in reference 24. The procedure was briefly summarized as followed. A pyrogallol solution is firstly mixed with Tris-HCl buffer at pH = 7.4 and the absorption at wavelength of 325 nm A (325 nm) is measured every 30 s within 5 minutes. We obtained theΔ*A*_control_ using this equation: Δ*A*_control_ = *A*_325nm,300s_ − *A*_325nm,30s_. Second, the sample solution was mixed with Tris-HCl buffer containing pyrogallol solution and the *A*_325nm_ was also obtained every 30 s within 5 minutes. We obtained the Δ*A*_sample_ using this equation: Δ*A*_sample_ = *A*_325nm,300s_ − *A*_325nm,30s_. Then, O_2_^**.–**^**-**scavenging activity of the sample was estimated according to the following equation:


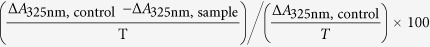


T = 5 min in this equation. The concentration for 50% O_2_^**.–**^ inhibition was defined as the IC_50_ value of the sample. All values represent three independent experiments and are expressed as the mean ± S.D. The Statistical analysis was performed using the SPSS software.

#### Fluorescence spectroscopy analysis

Aβ(1–42) was dissolved by brief vortexing in a 100% dimethyl sulfoxide (DMSO) solution at a concentration of 100 μM and stored at –80 °C. Just before assaying, the fresh Aβ(1–42) solution was sonicated for 10 min at 4 °C and then diluted to the indicated concentration (40 μM or 60 μM) in 20 mM PBS, 100 mM NaCl (pH = 6.0). Stocks of curcumin and ferulic acid (1 mM) were dissolved in 100% ethanol, filtered and stored at −80 °C. Just before use, curcumin and ferulic acid were diluted to the indicated concentration (40 μM or 60 μM) in 20 mM PBS, 100mM NaCl (pH = 6.0). In addition, 40 μM or 60 μM curcumin in 20 mM PBS, 100 mM NaCl (pH = 9.0) were heated for 20 min at 80 °C. After the temperature was down, the pH was adjusted to 6.0, and this solution represented the degradation products mixture of curcumin. A 60 μM stock of ThT was prepared in PBS buffer immediately before use.

A fluorescence spectroscopic study was performed on a Hitachi F-4500 fluorescence spectrophotometer. Briefly, each volumes of ThT solution and Aβ(1–42) with PBS buffer and curcumin or degradation products mixture of curcumin or ferulic acid, were mixed in a 1:1:1 volume ratio, and then incubated at 37 °C for 6 h. The final concentrations of Aβ(1–42), ThT, curcumin, curcumin to generate the degradation products mixture, and ferulic acid were all 20 μM in these experiments. Blank samples with PBS buffer and 20 μM ThT alone were analyzed to correct for background fluorescence by subtraction. The fluorescence spectroscopy experiments were carried out at the excitation and emission wavelengths of 440 and 485 nm. Each spectrum was the average of three independent scans. Error bar indicated standard deviations.

#### Electron microscopy analysis

To prepare specimens for EM analysis, 40 μM Aβ(1–42) was mixed with a 1:1 volume ratio with PBS buffer, or the same concentrations of curcumin, degradation products mixture of curcumin, or ferulic acid in Eppendorf tubes and incubated for 6 h at 37 °C. At the end of incubation, an aliquot (4 μl) of each reaction from each tube was immediately absorbed onto glow-discharged carbon-coated butwar films on 400-mesh copper grids. After 30 s, excess sample was blotted, and the grid was washed with 2% (W/V) uranyl acetate and negatively stained for 30 s. Samples were dried and transferred into a Tecnai G2 Spirit BIOTWIN Electron microscope (FEI) operated at 120 kV. Images were recorded with a Gatan 895 4k × 4k CCD camera at a nominal magnification of 30,000 ×.

#### Theoretical methods

To ensure the accuracy of the docking calculations, besides AChE and BACE-1, other six eligible enzymes met the following two inclusion criteria: i) with experimentally determined inhibitory activities of curcumin^46^,^47^,^48^,^49^,^50^,^51^; and ii) released crystal structures, were employed as model systems after reviewing the relevant studies. These six enzymes included HiV-1 PR, HiV-2 PR, ALR1, ALR2, COX-2 and SERCA. The crystal structures of AChE (PDB entry: 1B41)^52^, BACE-1 (PDB entry: 4XXS)^53^, HiV-1 PR (1HSG)^54^, HiV-2 PR (3EBZ)^55^, ALR1 (3H4G)^56^, ALR2 (1PWM)^57^, COX-2 (1CVU)^58^ and SERCA (3TLM)^59^ were obtained from the Protein Data Bank (PDB). The structures of the eight enzymes were compiled for docking calculations, respectively. All small molecules were firstly removed from the crystal structures of these enzymes. Then, all bonds in each enzyme were modified automatically and missing hydrogen atoms were added using Builder module in Insight II software package^60^. The partial atomic charges were assigned to each structure using CVFF force field using Discover Module in Insight II. The 3D structures of parent curcumin, its metabolites *in vivo*, its degradation products, and four reference compounds (physostigmine, indinavir, epalrestat and celecoxib) were first constructed using standard geometric parameters of SYBYL software package^61^, then were optimized using 1000 steps of steepest descent, followed by conjugate gradient minimization to a root mean square (RMS) energy gradient of 0.001 kJ/mol. Tripos force field and Gasteiger–Hückel charges were employed throughout the calculations.

The Surflex-Dock algorithm in SYBYL software was used for the molecular docking procedure, which offers the fully automated docking of flexible ligands to protein binding sites^62^. In recent years, Surflex-Dock are considered as the most accurate in terms of docking and it is clearly superior to other methods within the set of cases for which comparative data are available, with roughly double the screening enrichment performance^63^. The algorithm generates a pseudo-binding site called “protomol” as a target to generate putative poses of molecules^64^,^65^. Then, the putative poses are scored using the Hammerhead scoring function, which contains the hydrophobic contact and polar contact terms which are the dominant ones as well as the repulsive, entropic and solvation terms that possess lower contributions. The Hammerhead scoring function estimates binding affinities of a ligand-receptor complex in units of –log_10_K_d_, with an expected mean error of 1.0 log unit based on cross-validation. The function is differentiable with respect to ligand pose, so it is possible to refine a pose by performing gradient descent on the score, from a starting point to the optimal pose. In the present study, the ligand-based mode or automatic mode was adopted to generate the “protomol”, setting the Threshold and Bloat parameters as default value (0.5 and 0.0, respectively). Other parameters were adopted by default values in the Surflex-dock and no constraints were used for the molecular docking procedure. The maximum number of poses per ligand was set to 20.

## Additional Information

**How to cite this article**: Shen, L. *et al.* How does curcumin work with poor bioavailability? Clues from experimental and theoretical studies. *Sci. Rep.* **6**, 20872; doi: 10.1038/srep20872 (2016).

## Figures and Tables

**Table 1:** Theoretically estimated −log_10_K_d_ of four reference molecules and their experimental IC_50_ (μM) for six enzymes.

Enzymes	Physostigmine		Indinavir		Epalrestat		Celecoxib	
	−log_10_K_d_	IC_50_^a^	−log_10_K_d_	IC_50_^a^	−log_10_K_d_	IC_50_^a^	−log_10_K_d_	IC_50_^a^
AChE	7.319	0.041^33^						
HiV-1 PR			7.699	0.023^34^				
HiV-2 PR			7.523	0.041^34^				
ALR1					4.975	12.5^35^		
ALR2					5.745	1.4^35^		
COX-2							7.222	0.04^36^

^a^Experimentally determined IC_50_.

**Table 2:** Theoretically predicted IC_50_ (μM) of curcumin, its *in vivo* metabolites, and degradation products for eight model enzymes.

**Inhibitors**	**AChE**	**BACE-1**	**HiV-1 PR**	**HiV-2 PR**	**ALR1**	**ALR2**	**COX-2**	**SERCA**
Experimental IC_50_ using curcumin as inhibitor	67.69^46^	340^47^	100^48^	250^48^	>200^49^	10^49^	35^50^	7~15^51^
Curcumin (keto)	0.15	0.07	0.13	0.17	1.0	0.08	1.58	0.03
Curcumin (enol)	0.09	0.36	0.22	0.28	5.1	0.09	1.66	0.01
Tetrahydrocurcumin	1.86	1.20	0.91	1.69	0.81	1.32	0.15	0.05
Hexahydrocurcumin	1.25	0.98	0.22	1.82	0.32	0.69	0.21	0.02
Octahydrocurcuminol	0.68	1.79	0.06	0.12	0.30	0.64	0.41	0.01
Trans-6-(4′-hydroxy-3′-methoxy-phenyl)-2,4-dioxo-5-hexenal	12.5	96.5	14.5	16.5	57.5	11.4	36.3	0.5
Feruloyl methane	47.9	165	12.6	24.5	87.1	5.4	177.8	11.7
Ferulic acid	60.7 (128.7)^a^,^32^	199	21.4	275	114	65 (61.4)^a^,^33^	30.2	14.5
Ferulic aldehyde	23.2	252	117	38.9	105	124	30.6	15.1
Vanillin	60.4	473	372	158	549	157	37.2	91.2

^a^Experimental values using ferulic acid as inhibitor in parentheses.
